# Nurses’ perceptions of a digital supportive intervention for patients with breast cancer receiving neoadjuvant chemotherapy: a qualitative study

**DOI:** 10.1177/17449871261426285

**Published:** 2026-03-25

**Authors:** Kristina Rooth, Kay Sundberg, Linda Gellerstedt, Tina Gustavell, Ann Langius-Eklöf, Maria Fjell

**Affiliations:** MSc, Department of Neurobiology, Care Sciences and Society, Division of Nursing, Karolinska Institutet, Sweden; Associate Professor, Department of Neurobiology, Care Sciences and Society, Division of Nursing, Karolinska Institutet, Sweden; PhD, Department of Neurobiology, Care Sciences and Society, Division of Nursing, Karolinska Institutet, Sweden; PhD, Department of Neurobiology, Care Sciences and Society, Division of Nursing, Karolinska Institutet, Sweden, and Karolinska University Hospital, Cancer Theme, Department of Upper Abdominal Diseases, Sweden; Professor, Department of Neurobiology, Care Sciences and Society, Division of Nursing, Karolinska Institutet, Sweden; PhD, Department of Neurobiology, Care Sciences and Society, Division of Nursing, Karolinska Institutet, Sweden

**Keywords:** breast cancer, digital intervention, ePRO, neoadjuvant chemotherapy, nurse support, symptom management

## Abstract

**Background:**

Interventions using electronic patient-reported outcomes (ePROs) have proven effective in enhancing patient support and self-care during cancer treatment. Successful implementation depends on nurses’ acceptance, as they play an important role in patient care and support.

**Aim:**

To describe nurses’ perceptions of an interactive digital intervention (Interaktor) to provide support in symptom management for patients with breast cancer during neoadjuvant chemotherapy.

**Method:**

This qualitative study involves paired interviews with four purposively sampled nurses involved in a complex digital support intervention at two oncology clinics in Sweden. Data were analysed using reflexive thematic analysis.

**Results:**

Two themes reveal that Interaktor can be effectively integrated into clinical practice, highlighting the importance of establishing routines, allocating time, and customising user-friendliness. The nurses were enabled to identify patients’ needs for additional support and enhance their ability to involve patients in their care. Potential benefits for other healthcare professionals were recognised.

**Conclusions:**

The integration of Interaktor into breast cancer care was accepted by nurses and may offer benefits for patients and healthcare professionals. Using ePROs can help identify patients with an extended need for supportive care. Time constraints, digital skills, and workflow integration are challenges that need to be addressed for future implementation.

## Introduction

Breast cancer is the most common cancer among women worldwide (Bray et al., 2024). The treatment can include surgery, chemotherapy, radiation therapy, targeted drugs, and endocrine therapy (Burguin et al., 2021). Tumours with more aggressive characteristics are often treated before surgery with neoadjuvant chemotherapy (NACT) (Confederation of Regional Cancer Centres in Sweden, 2025; Riis 2020). Chemotherapy can cause severe physical symptoms such as nausea and joint pain, as well as psychological concerns such as worry and anxiety (Liu et al., 2021). Patients with breast cancer often remain at home between treatments since it is usually outpatient-based (Confederation of Regional Cancer Centres in Sweden, 2025; Lai et al., 2017). This may result in patients having to manage their symptoms and perform self-care independently for a long period (Lai et al., 2017). Patients have identified information about self-care and treatment, family and emotional support, and encouragement from healthcare professionals as important care needs (Liu et al., 2021).

The use of digital interventions in healthcare is rapidly increasing (Ardito et al., 2023) and is associated with high patient satisfaction and acceptance (Gyawali et al., 2023). Collecting electronic patient-reported outcomes (ePROs) during cancer treatment has been shown to improve health-related quality of life (Balitsky et al., 2024), symptom control (Marthick et al., 2021; Singleton et al., 2022), and survival (Balitsky et al., 2024) compared to control groups. Moreover, ePRO-based symptom monitoring and communication in cancer care have been shown to be safe and supportive to patients (Kaye et al., 2020). Patients with cancer generally perceive digital interventions positively, particularly for accessing symptom and illness-related information. However, they emphasise the importance of complementing these interventions with personal contact from healthcare professionals (Jansen et al., 2015). Healthcare professionals have raised concerns about increased workload, data privacy, and reduced communication between them and patients as barriers to implementing digital interventions (Ardito et al., 2023).

The Interaktor app used in the present study is an interactive digital platform co-designed between patients and healthcare professionals (Langius-Eklöf et al., 2017). It was developed to support patients with cancer by enabling real-time ePRO collection, providing guidance for managing symptoms and concerns, and facilitating interaction with a nurse. Through the app, patients regularly report symptoms and have continuous access to self-care advice and graphs of their reported symptom history. A free-text message can be included before submitting their report. The effects of using Interaktor on patient outcomes during treatment for breast, prostate, and pancreatic cancer have previously been evaluated and show positive results regarding symptom burden and adherence (Fjell et al., 2020; Gustavell et al., 2019; Kelmendi et al., 2025; Sundberg et al., 2017). Furthermore, patients have perceived using Interaktor as reassuring and supportive during treatment (Crafoord et al., 2020; Gustavell et al., 2020; Kelmendi et al., 2025). To date, our research has focused on patient acceptability and benefits, and less on how nurses, who work closely with patients in their care, perceive Interaktor. It is emphasised that knowledge about how digital interventions work in clinical practice and the acceptance of healthcare professionals is essential for future development and implementation (Wernhart et al., 2019). There is a high probability that benefits for patients may not be achieved if healthcare professionals are not comfortable using digital systems (Huter et al., 2020). Therefore, it is essential to understand how Interaktor can be further developed to facilitate interaction between nurses and patients. This study aims to describe nurses’ perceptions of an interactive digital intervention (Interaktor) to provide support in symptom management for patients with breast cancer during neoadjuvant chemotherapy.

## Methods

### Design

This study has a descriptive qualitative design and is part of an evaluation of a complex intervention of digital support during treatment for breast cancer (Langius-Eklöf et al., 2017). It consists of interviews with nurses who were part of the intervention in a randomised controlled trial (RCT) underpinned by the Medical Research Council’s framework for complex interventions (Craig et al., 2008). The 32-item checklist for reporting qualitative research (COREQ) was used to report this study (Tong et al., 2007) and is included in the Supplemental Material.

### Setting

The intervention with Interaktor was conducted at the oncology clinics of two university hospitals in Region Stockholm, Sweden. Patients were included between June 2015 and March 2017. The RCT included patients with breast cancer undergoing NACT.

Standard care in Region Stockholm during NACT at the time of the RCT entailed visits to the physician at the oncology clinic before each treatment cycle. Depending on the chemotherapy regimen, the visits occurred approximately every second or third week. Patients are assigned a contact nurse when receiving a cancer diagnosis. A contact nurse has advanced specialist education in oncology. Their role is to facilitate communication between healthcare services and patients, and support throughout the treatment trajectory (Confederation of Regional Cancer Centres in Sweden, 2019). Patients in this study had an appointment with their assigned contact nurse within a few days following their initial consultation with the physician. Subsequently, the contact nurse conducted a follow-up phone call with the patient after their first treatment cycle. Beyond this point, there were no scheduled routine interactions with the contact nurse during NACT.

### The intervention Interaktor

Interaktor for NACT during breast cancer consists of two components: an app for patients to report ePROs and a web interface for nurses (Fjell et al., 2020). Displayed in the web interface, the nurses could monitor reports in real-time from patients on the occurrence, frequency, and distress level of 14 common symptoms during NACT. Reports were sent from the first day of NACT until 2 weeks after completion, with an average reporting duration of 18 weeks. Patients were instructed to report their symptoms daily, Monday to Friday, between 8:00 AM and 4:00 PM. The risk assessment model for symptoms that triggered alerts sent a text message containing a code representing each patient to a study-specific smartphone at the clinics. In case of an alert, nurses contacted the patient by phone to discuss the symptoms and their management. There were two types of alerts, predetermined based on symptom severity: yellow and red. In the event of a yellow alert, nurses were expected to contact the patient during the day. For red alerts, nurses were expected to contact the patient within 1 hour, any actions taken could be documented in the web interface. Over the nearly 2-year study period, 74 patients used Interaktor. Their reports generated a total of 1,276 alerts: 1,094 were yellow alerts, and 182 were red. Illustrations and more details about the app have previously been published (Fjell, 2021). All data were stored on a secure server.

Before the start of the RCT, nurses at the clinics were individually introduced to Interaktor. They received written and verbal information as well as a study folder. Using fictive patients, the nurses could familiarise themselves with the web interface. During the study, technical support was available via phone and email.

### Sample

Four female contact nurses who were involved throughout the entire study period agreed to participate in two paired interviews, one at each clinic. Nurses who only intermittently handled phone alerts chose not to participate in the interviews. Paired interviews were selected because the interaction between participants can reveal nuances that might not emerge in individual interviews (Wilson et al., 2016). The researchers obtained permission from the managers at the clinics to contact the nurses via email.

### Data collection

In February 2018, the interviews were conducted face to face in conference rooms at the two clinics where the nurses worked. The interviews were facilitated by a moderator and an observer, both of whom were female healthcare professionals and members of the same research team as the authors. Both interviewers had prior experience in conducting research interviews and engaging with patients in healthcare settings. Neither had any personal or professional relationship with the study participants. The semi-structured interview guide, developed by the research team, focused on implementation, usage, patient contact, impact on the work environment, and functions within the web interface, including alerts, free-text function, self-care advice, history of reported symptoms, and the approach in general (Textbox 1). Both interviews started with the moderator summarising the intervention, and if needed, probing questions were asked. The interviews lasted 54 and 63 minutes, respectively, and were audio-recorded with the participants’ permission.

**Textbox 1.** Main questions in the interview guide.

• Can you describe how the study-specific phone and the alerts were managed? • What is your perception of the usability of the intervention? • How have the reports influenced the contact with the patients who used the app? • How has the way of working been affected by the intervention? • What meaning do you think the self-care advice in the app had? • What are your views and reflections about introducing this kind of approach? • Do you have any suggestions for changes or improvements to the intervention?

### Data analysis

The audio-recorded interviews were transcribed verbatim by a professional transcription service external to the research team and subsequently checked for accuracy by the first author. All data were collected before the analysis began and analysed with an inductive approach using reflexive thematic analysis as described by Braun and Clarke (2021). The authors strived for reflexivity by consistently reflecting on their expectations, assumptions, choices, actions, and roles as researchers and nurses throughout the analysis. Furthermore, by keeping reflexive notes, the authors could gather and revisit thoughts and reflections throughout the analysis. The 15-point checklist of criteria for good thematic analysis was followed for a rigorous, systematic and reflexive analysis (Braun and Clarke, 2021).

**Figure 1.**
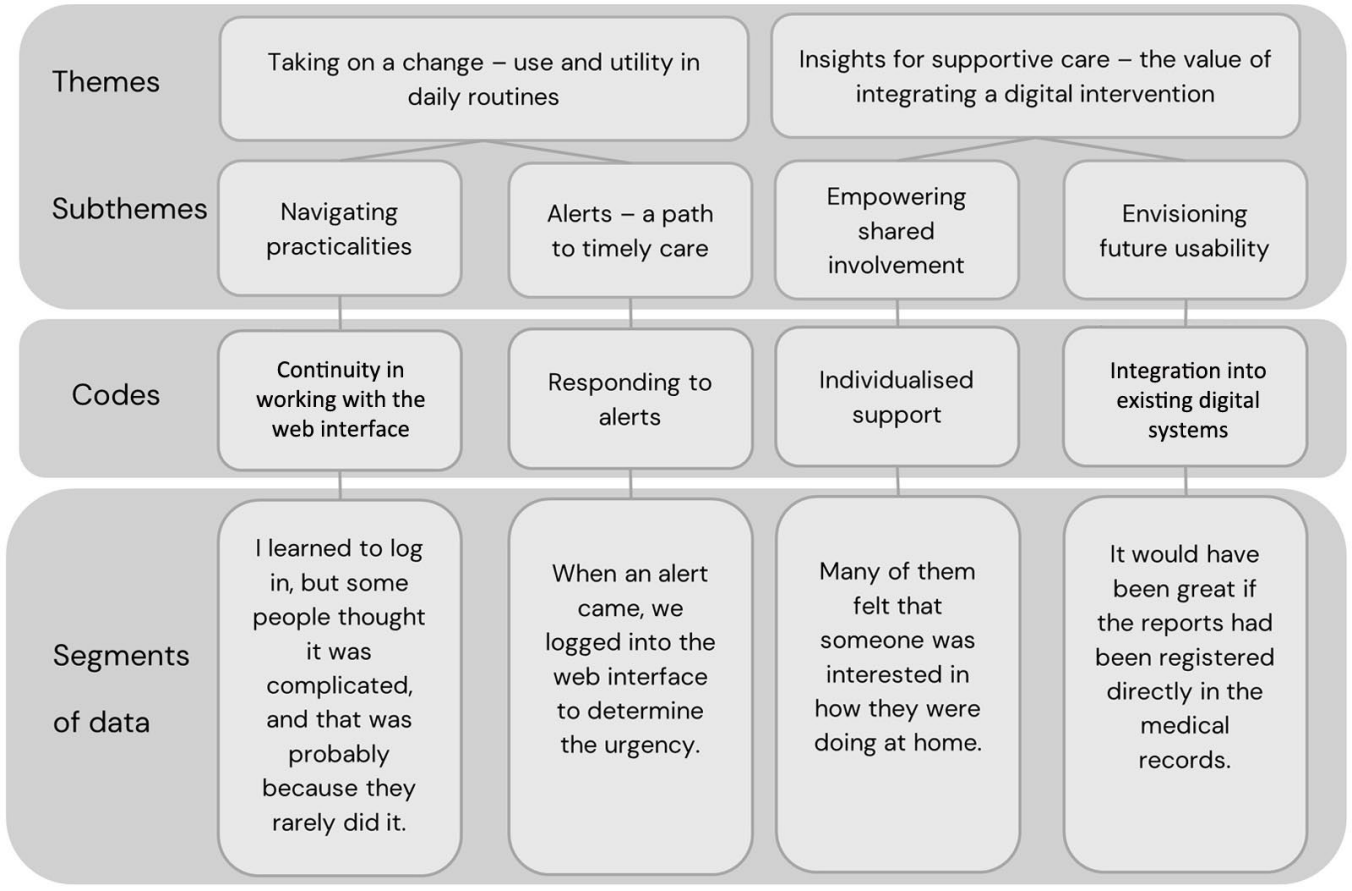
Illustrative summary of the analytic process.

In the first phase of the analysis, the first and last authors separately listened to the audio recordings and read the transcripts repeatedly to become familiar with the dataset. Notes with initial ideas for coding were made. In the second phase, segments of data were identified in alignment with the aim and transferred to a Microsoft Excel^®^ file. The segments of data, coded by the first and last author, ranged from a few words to complete sentences. Through an interpretive process involving several meetings, the codes were presented to all authors, developed and decided upon. In the third phase, initial themes were constructed as the first and last authors identified shared patterns of meaning within the dataset. Codes that aligned with each other were then collated into four themes in a thematic map. In the fourth phase, all authors discussed and revised the themes on several occasions until consensus was reached upon two themes and four subthemes. The themes and subthemes were further developed in phase five by refining, defining and naming them. In the sixth phase, all authors contributed to the final analysis and writing of the results. For an illustration of the analytic process, see Figure 1.

### Ethical considerations

Ethical approval for the study was obtained from the Swedish Ethical Review Authority (Dnr: 2013/1652-31/2 and 201712519-32), and the study was conducted following the Declaration of Helsinki (World Medical Association, 2013). All participants received both written and verbal information about the study before inclusion. The voluntary nature of participation was emphasised, and the right to withdraw at any moment without giving a reason was explained. Participants were informed that quotes from the interviews would be formulated to prevent individuals from being identified.

### Trustworthiness

Credibility was supported through repeated readings, regular analytic discussions among the authors and constant comparison of interpretations with the data. Transferability was ensured by providing a detailed description of the study context and sampling strategy. An audit trail offered a transparent and thorough account of the analytic process, thereby strengthening dependability. Confirmability was promoted through reflexive practices, as the authors reflected on their prior knowledge and their roles as nurses and researchers (Lincoln and Guba, 1985).

## Results

Nurses 1 and 2 (referred to in the findings as pair A) worked at one of the clinics, and nurses 3 and 4 (pair B) worked at the other.

### Taking on a change: use and utility in daily routines

#### Navigating practicalities

The nurses described how each day started with assigning which nurse would be responsible for the study-specific phone. This nurse would then inform the patient’s contact nurse in case of an alert, an approach which provided patients with continuity during treatment. In times of high workload, the nurses collaborated to phone patients in response to incoming alerts.

The nurses voiced that the ability to log in to the web interface varied among the nurses involved during the study period.

Some needed help to log in to the web interface, while others did not encounter any problems. (Nurse 4, pair B)

They noted that discomfort with using the web interface could create frustration and reduce motivation for some nurses, leading to taking less responsibility. Those who experienced no difficulties found it enjoyable to use.

The nurses perceived that they did not have enough time to familiarise themselves with Interaktor. They reflected on the challenge of monitoring the web interface without affecting their normal work tasks. Despite this, they deemed Interaktor a great and timely tool that could be helpful in recognising patients in need of support.

This tool (Interaktor) generates important information, which could help us a lot, but we had far too little time to work with it. (Nurse 3, pair B)

It was observed that patients seemed to enjoy using Interaktor, and their positive response gave the nurses an optimistic attitude towards future implementation. They believed it could be effectively integrated into their daily work routines, although they had a few suggestions to enhance user-friendliness. Suggestions included muting the colours, placing the free-text messages more visibly, and making it easier to identify patients in the scroll list, as their names were coded. Establishing routines and ensuring continuity were identified as key factors for implementing an intervention such as Interaktor, without being too burdensome.

#### Alerts: a path to timely care

There were variations in the frequency and type of alerts generated from the reports; some patients never had alerting symptoms, whereas others did frequently. Reports from patients receiving dose-dense treatments triggered more alerts, leading to more frequent contact. According to the nurses, the most frequently generated alerts concerned fever, nausea, and oral problems. When an alert came, they logged in to the web interface and the patient’s medical records to prioritise the urgency of the alerts. The focus was to determine the patients’ current stage in the treatment cycle and the time of their next scheduled visit to the physician.

We always looked in the medical record to prepare for the phone call with the patient and to see if there were any recent test results. (Nurse 1, pair A)

If the alert is fever just before a chemotherapy session, the blood cells are usually not alarmingly low, but if it is fever a few days after a session, then you think ohohoh and might want a blood sample quickly to check the levels of blood cells. (Nurse 2, pair A)

When an alert was generated, nurses sometimes wished for more information in the web interface to be better prepared before calling the patients. Additional details, such as symptom duration, ongoing medication, or whether the patient experienced any other symptoms, would have been helpful. The phone calls provided the patients with confirmation that the nurse had received and acknowledged incoming alerts.

The alerts generated from the reports gave patients a sense of reassurance, because we responded by phone. (Nurse 1, pair A)

Alerts were generally not perceived as stressful, except for the requirement to respond to red alerts within 1 hour, a target the nurses occasionally failed to meet. Contributing factors to these delays included time constraints, the inability to interrupt ongoing tasks, scheduled appointments with other patients, or staffing shortages. The nurses also described how a patient’s single report could generate multiple alerts.

When patients had more than one severe symptom, several alerts were generated from one report, but usually only required us to call them once. (Nurse 2, pair A)

A challenge was that some patients sent reports beyond the study time frame, such as late at night or during weekends. This led to unanswered alerts the following morning, causing uncertainty about whether patients needed to be contacted or if they had already been in touch with the healthcare service.

Some patients whose symptoms triggered alerts learned to manage them by themselves and asked the nurses not to contact them for each alert. In these cases, the nurse and patients agreed that the patients would use a free-text message to communicate this preference.

### Insights for supportive care: the value of integrating a digital intervention

#### Empowering shared involvement

The nurses observed that the reports had varying meanings for patients. They could serve as a reminder, since patients easily forget how they felt days or weeks earlier, or as a convenient way to contact the clinic when they lacked the energy to call. Another meaning was that using Interaktor made the patients feel seen by the nurses. This was interpreted as patients perceiving that someone cared about their health and that they were actively involved in their care.

By reporting in Interaktor, patients felt like they were in the system, that someone saw them and that they were not forgotten. (Nurse 3, pair B)

The nurses acknowledged that patients used free-text messaging to elaborate on their well-being and to share details about their daily lives at home. Patients’ continuous access to information and guidance provided in the self-care advice was a valuable complement to standard written materials.

I think the continuous access to the self-care advice in the app is really good, so patients can read on the subway or whenever there is a need. So, of course, it is a great thing for them. (Nurse 1, pair A)

They believed patients were empowered by being able to take action themselves to alleviate symptoms or concerns. The nurses found the various self-care advice well-written but suggested that some could be condensed to make it less overwhelming for patients. Overall, they perceived that patients who used the app had fewer questions when interacting with the nurses at the oncology clinic.

Ordinarily, we inform patients about constipation on several occasions, but when they could read about it at home, I noticed it was rarely brought up during the visits. (Nurse 4, pair B)

The nurses considered the reports informative and frequently used them to prepare for scheduled patient visits. The graphs displaying the history of reported symptoms enabled the nurses to monitor patients’ symptom development over time.

#### Envisioning future usability

During the interviews, they discussed how access to the history of reported symptoms could be helpful for other healthcare professionals in supporting patients.

The physician could use the history of reported symptoms to evaluate the potential effects of modifications in medication or treatment plans. (Nurse 2, pair A)

Nurses expressed concerns that patients with limited proficiency in Swedish might experience difficulties using Interaktor. Even without language barriers, a system like this can be challenging for patients to navigate in an already stressful situation. Based on their experiences, patients have varying needs regarding their care. Some patients prefer information on a need-to-know basis, whereas others want more individual support.

The aim should be to reach as many as possible, but without imposing it (Interaktor) on anyone who does not feel comfortable with it. (Nurse 3, pair B)

They discussed whether age might influence patients’ comfort with digital technology. Observations indicated that older patients who participated in the study appeared to enjoy using the app, highlighting the importance of avoiding preconceptions. They also noted that others, such as close relatives who frequently accompany patients to clinic visits, could also benefit from using Interaktor.

## Discussion

This study offers valuable insights from nurses on the use of an interactive digital intervention during NACT for patients with breast cancer. Nurses recognised the potential of integrating an app like Interaktor into clinical practice. They highlighted its role in facilitating the detection of alarming symptoms between chemotherapy cycles, enabling continuous symptom monitoring, and providing patients with tailored advice for managing severe symptoms. Furthermore, nurses perceived that patients demonstrated a positive attitude towards the app and benefited from its use.

The nurses regarded monitoring incoming alerts as a daily routine to help identify patients’ needs for support and enhance interaction with them. The additional information about patients’ well-being helped them prioritise the urgency of concerns and prepare for patient visits to provide individual support. This result is consistent with previous research, which has shown that regular reporting of ePROs improves clinical decision-making, enhances patient-centred care, and enables individualised support (Balitsky et al., 2024; Di Maio et al., 2022). Digital interventions have also been shown to have a positive effect on communication with healthcare professionals (Gyawali et al., 2023). However, nurses have raised concerns about relying solely on digital reports from patients and emphasised the importance of personal interaction (Shelley et al., 2024). Studies have demonstrated that digital interventions could be a supplementary resource to personal interaction with healthcare professionals (Hallberg and Salimi, 2020; Troncoso and Breads, 2021).

The results from the current study show that the nurses considered Interaktor suitable for daily practice, but they pointed out some factors to reflect on before implementation. They identified a lack of time for learning and implementation. Establishing routines before implementing digital systems has been emphasised (Frennert et al., 2023; Fridriksdottir et al., 2023). Another important factor discussed in the interviews was that some colleagues were reluctant to use Interaktor due to login difficulties. The digital literacy of healthcare professionals needs to be considered when implementing digital interventions (Ekstedt et al., 2023; Kemp et al., 2021). Super users or champions at the workplace have been recognised as preferable by healthcare professionals when implementing digital interventions (Cheung et al., 2022).

There was a suggestion to integrate the web interface with the medical records, allowing other healthcare professionals to use the reports to evaluate treatment outcomes and prepare for patient visits. Automatic entry of ePROs into the medical record can improve patient safety (Borkar et al., 2024). Another area of development was providing Interaktor in languages other than Swedish. Language disparities have been recognised as a barrier in utilising digital health interventions and can contribute to health inequity (Azizi et al., 2024).

The nurses perceived that Interaktor provided patients with a sense of reassurance and involvement in their care, while also enabling self-care. Interaktor allowed nurses to identify patients’ supportive needs in real time, facilitating self-care or initiating physician referrals based on symptom severity. These findings are consistent with previous findings on Interaktor during treatment for pancreatic cancer (Mangsbacka and Gustavell, 2022) and other comparable interactive digital interventions in cancer care (Fridriksdottir et al., 2023; McCann et al., 2024).

Collectively, these findings suggest that integrating a digital intervention such as Interaktor into standard care can be acceptable for nurses to facilitate communication and support during cancer treatment. Strategies for successful implementation should include dedicated time and support for training and use, established routines, and integration with existing medical records to optimise workflow. Although this study focused on the treatment phase, few nurse-led digital interventions in breast cancer care have targeted the follow-up period (Brown et al., 2021). As some patients express a need for continued support from healthcare professionals after treatment (Khajoei et al., 2023; Rooth et al., 2024), future research should explore the potential benefits of Interaktor during the follow-up period.

### Strengths and limitations

The interviewed nurses were those most actively engaged with the intervention. This close involvement enabled them to provide detailed, experience-based insights. The sample size could introduce a risk of selection bias and affect the trustworthiness and rigour. Albeit, sample size is not solely determined by the number of participants, but by the ability of the data to offer a nuanced and in-depth understanding of the phenomenon under study (Hennink and Kaiser, 2022). Although paired interviews enriched the data through shared experiences and reflective dialogues, the format may also have influenced responses due to group dynamics (Wilson et al., 2016). Demographic information on the nurses could have been beneficial; however, such data were not collected because it fell outside the scope of the ethical approval. A further limitation was that the interviews took place nearly 6 months after the intervention, which may have affected the nurses’ ability to remember specific details. However, interviewing two participants at a time may have facilitated memory recall.

## Conclusion

This study shows that working with a digital supportive care intervention was acceptable for nurses in cancer care, and they could recognise the advantages for patients. The use of ePROs can facilitate the identification of patients with an extended need for supportive care. There is a potential for integrating supportive care interventions into daily routines; however, nurses’ time constraints, digital skills, and existing working practices must be acknowledged as key factors in future implementation.

**Key points for policy, practice, and/or research**

- Reports generating alerts enabled nurses to initiate timely care and identify patients with an extended need for support.
- Nurses perceived that the digital intervention Interaktor could be effectively integrated into daily routines at the oncology clinics, although allocated time, digital skills, and workflow integration were identified as barriers.
- Physicians could also benefit from the information provided by the reports when interacting with patients.
- Interaction with nurses between cancer treatment cycles was perceived as reassuring and enhancing communication to identify individual support needs.

## Supplemental Material

sj-pdf-1-jrn-10.1177_17449871261426285 – Supplemental material for Nurses’ perceptions of a digital supportive intervention for patients with breast cancer receiving neoadjuvant chemotherapy: a qualitative study Supplemental material, sj-pdf-1-jrn-10.1177_17449871261426285 for Nurses’ perceptions of a digital supportive intervention for patients with breast cancer receiving neoadjuvant chemotherapy: a qualitative study by Kristina Rooth, Kay Sundberg, Linda Gellerstedt, Tina Gustavell, Ann Langius-Eklöf and Maria Fjell in Journal of Research in Nursing
